# Relationships between Phyllosphere Bacterial Communities and Leaf Functional Traits in a Temperate Forest

**DOI:** 10.3390/plants12223854

**Published:** 2023-11-15

**Authors:** Zuoqiang Yuan, Ji Ye, Fei Lin, Xing Wang, Teng Yang, Boyuan Bi, Zikun Mao, Shuai Fang, Xugao Wang, Zhanqing Hao, Arshad Ali

**Affiliations:** 1 Shaanxi Key Laboratory of Qinling Ecological Intelligent Monitoring and Protection, School of Ecology and Environment, Northwestern Polytechnical University, Xi’an 710072, China; zqyuan@nwpu.edu.cn (Z.Y.); bby0710@nwpu.edu.cn (B.B.);; 2 CAS Key Laboratory of Forest Ecology and Management, Institute of Applied Ecology, Chinese Academy of Sciences, Shenyang 110016, China; yeji1011@163.com (J.Y.); wangxg@iae.ac.cn (X.W.); 3 Plant Ecology and Nature Conservation, Wageningen University & Research, 6708 PB Wageningen, The Netherlands; 4 State Key Laboratory of Soil and Sustainable Agriculture, Institute of Soil Science, Chinese Academy of Sciences, East Beijing Road 71, Nanjing 210008, China; tyang@issas.ac.cn; 5 Key Laboratory of Terrestrial Ecosystem Carbon Neutrality, Institute of Applied Ecology, Chinese Academy of Sciences, Shenyang 110016, China; 6 Forest Ecology Research Group, College of Life Sciences, Hebei University, Baoding 071002, China; arshadforester@gmail.com

**Keywords:** phyllosphere bacteria, plant phylogeny, functional traits, interspecific variation, community structure

## Abstract

As a vital component of biodiversity, phyllosphere bacteria in forest canopy play a critical role in maintaining plant health and influencing the global biogeochemical cycle. There is limited research on the community structure of phyllosphere bacteria in natural forests, which creates a gap in our understanding of whether and/or how phyllosphere bacteria are connected to leaf traits of their host. In this study, we investigated the bacterial diversity and composition of the canopy leaves of six dominant tree species in deciduous broad-leaved forests in northeastern China, using high-throughput sequencing. We then compare the differences in phyllosphere bacterial community structure and functional genes of dominant tree species. Fourteen key leaf functional traits of their host trees were also measured according to standard protocols to investigate the relationships between bacterial community composition and leaf functional traits. Our result suggested that tree species with closer evolutionary distances had similar phyllosphere microbial alpha diversity. The dominant phyla of phyllosphere bacteria were Proteobacteria, Actinobacteria, and Firmicutes. For these six tree species, the functional genes of phyllosphere bacteria were mainly involved in amino acid metabolism and carbohydrate metabolism processes. The redundancy and envfit analysis results showed that the functional traits relating to plant nutrient acquisition and resistance to diseases and pests (such as leaf area, isotope carbon content, and copper content) were the main factors influencing the community structure of phyllosphere bacteria. This study highlights the key role of plant interspecific genetic relationships and plant attributes in shaping phyllosphere bacterial diversity.

## 1. Introduction

The concept of phyllosphere was first introduced by a British Pathologist (named F.T. Last) in 1955 and is defined as the outer surface environment of leaves with complex microbial communities [1]. However, scientists have recently found a further connection between plant leaves’ outer surface microbial communities and their inner parts. For example, microbes can enter the leaves from the outer surface through the stomata and veins of plants [2]. Recent studies suggest that the surface area of all leaves may be twice as large as the global land surface area, making it a significant microbial habitat on Earth [3]. Most studies have traditionally focused on the surface of leaves, but in recent years, researchers have also begun to include the leaf margin (i.e., above-ground plant parts). This area is now recognized as providing a suitable living environment for certain microbes, according to studies such as those by Farre-Armengol et al. [4] and Stone Bram and Jackson Colin [5].

Phyllsophere microbes are essential in maintaining plant health and ecosystem functions [6,7,8]. Previous studies have found that the phyllosphere microorganism *Pseudomonas lurida* has an antagonistic effect on pathogenic microbes to maintain plant host health [9]. Further, the phyllosphere microorganism *Staphylococcus* can improve the adaptability of host plants to adversity due to the production of indole-3-acetic acid (IAA) [10]. As for factors affecting the composition of the phyllosphere microbial community, most studies argue that the type of host plants is important [11,12,13,14,15,16]. For example, bacterial community structure on leaves was highly correlated with host evolutionary relatedness and suites of plant functional traits related to host ecological strategies for resource uptake and growth/mortality tradeoffs, whereas the abundance of several bacterial taxa was correlated with host growth, mortality, and function [11,12,13,14,15,16]. 

Furthermore, plant functional traits could regulate microbial communities due to the lack of nutrients on the leaf surface [13,17,18,19]. Phyllosphere microbes rely on the host plant to obtain their required nutrients [20]. Therefore, the physiological characteristics of the host plant can determine whether the phyllosphere microbes can successfully colonize or not [13]. Studies have reported that leaf nitrogen and phosphorus elements strongly influence phyllosphere bacterial communities, which depend on leaf traits [19,21]. Moreover, aluminum and copper in the leaves of tropical forests in Panama are related to the function of phyllosphere bacteria, which is associated with the ability of these two elements to resist pests and diseases [17]. Nonetheless, in contrast to our understanding of rhizosphere microbes, the role of phyllosphere microbes within the environment, as well as the underlying interaction mechanisms between phyllosphere microbes and plants, remain largely uncharted.

The forest canopy is the main interface where forests interact with the external environment, and it is responsible for supporting a significant number of species on Earth [22,23,24]. The conservation and sustainable use of forest canopy biodiversity in the face of climate change is a crucial topic in ecological research and has been widely studied [22,25]. In this context, the broad-leaved Korean pine mixed forest, as the typical vegetation in temperate forests, is famous for its complex structure and unique species composition, in comparison to regions at the same latitude worldwide [26]. Although there are many studies on the interactions among animals, plants, and soil microbes in the study region [27,28,29], there is still weak knowledge of canopy phyllosphere bacteria which restricts our understanding of microbial composition and its impact on forest community structure and function in natural ecosystems [7]. This study aims to determine the main the community composition and and the driving factors of canopy phyllosphere bacteria in the temperate broad-leaved Korean pine mixed forest of Changbai Mountain in northeastern China. To do so, we collected canopy leaves of six dominant tree species to answer the following three questions: (1) what are the similarities and differences in the alpha diversity and community composition of phyllosphere bacteria in different tree species? (2) are there unique biomarkers for each tree species? and (3) are there potential associations between phyllosphere bacterial communities and leaf functional traits? We hypothesize that: (1) tree species with closer evolutionary distance should have similar alpha phyllosphere microbial diversity; (2) tree species do not have unique dominate phyla, as microbes generally communicate between long-term coexisting tree species; and (3) functional traits regulate microbial community composition.

## 2. Results

### 2.1. Phyllosphere Bacterial Diversity and Composition

**Figure 1:**
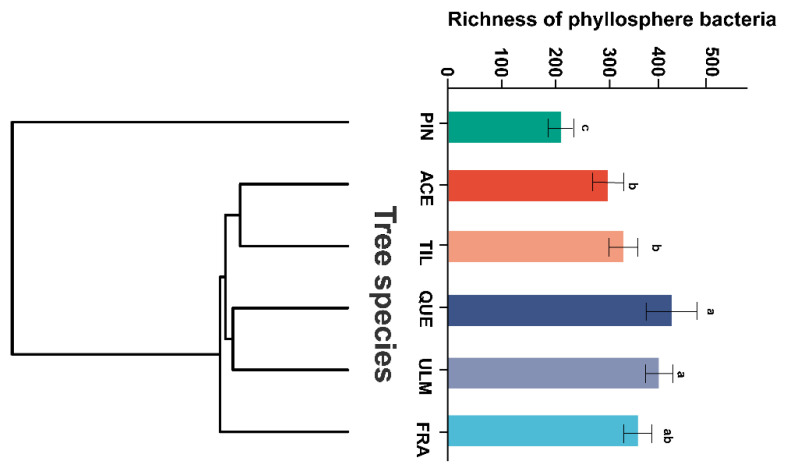
The richness of phyllosphere bacteria arranged according to tree’s phylogenetic relationships in a temperate mixed forest of Changbai Mountain. If the branches between two tree species are shorter, it signifies that they are closer in terms of evolution. Conversely, longer branches indicate a more distant genetic relationship. Letters “a”, “b”, and “c” represent distinct groups identified through ANOVA. Groups sharing the same letter signify no significant difference (*p* > 0.05), whereas groups with different letters indicate a significant difference (*p* < 0.05). Abbreviations of tree species: ACE: *Acer mono*; FRA: *Fraxinus mandshurica*; PIN: *Pinus koraiensis*; QUE: *Quercus mongolica*; TIL: *Tilia amurensis*; ULM: *Ulmus japonica*.

The phyllosphere bacterial richness exhibited significant variations among different tree species, with the highest richness observed in *Q. mongolica*, while *P. koraiensis* had notably lower bacterial richness compared to the other tree species. When comparing the phyllosphere bacterial diversity between *Q. mongolica* and *U. pumila*, no significant differences were detected. However, both tree species exhibited significant dissimilarities in bacterial diversity compared to the remaining tree species, except for *F. mandshurica* (Figure 1). Notably, bacterial richness exhibits significant differences among tree species with closer phylogenetic relationships, such as *A. mono* and *T. amurensis*, as well as *Q. mongolica* and *U. japonica*. In contrast, significant differences in bacterial richness were observed between host plants with more distantly related evolutionary backgrounds, for instance, *F. mandshurica* and *P. koraiensis*.

**Figure 2:**
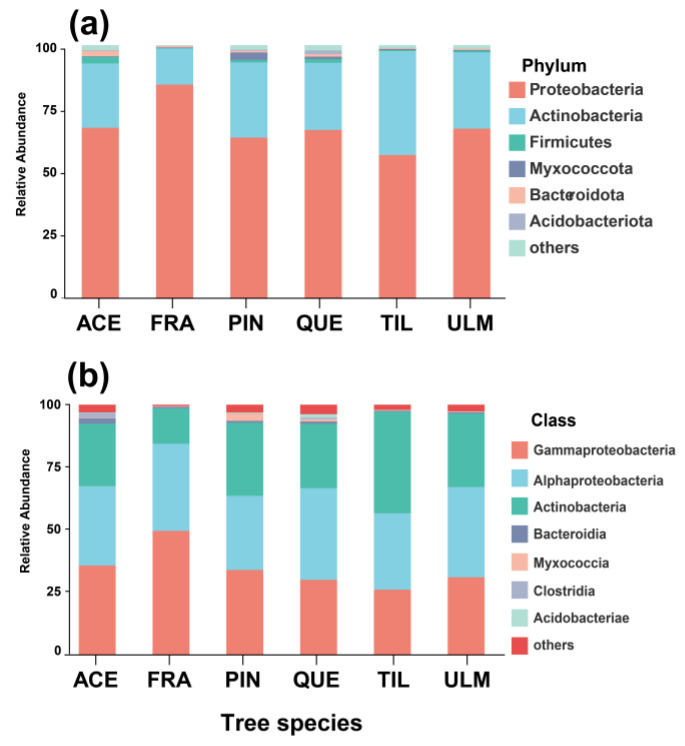
Histogram of relative abundance of phyllosphere bacteria of dominant tree species in a temperate mixed forest of Changbai Mountain at the level of phylum (**a**) and class (**b**) level. Abbreviations of tree species: ACE: *Acer mono*; FRA: *Fraxinus mandshurica*; PIN: *Pinus koraiensis*; QUE: *Quercus mongolica*; TIL: *Tilia amurensis*; ULM: *Ulmus japonica*.

A total of 11 phyla, 46 classes, 147 orders, 401 families, and 1077 genera were identified from the leaves of the 6 tree species studied. Proteobacteria (67.7%), Actinobacteria (27.8%), and Firmicutes (1.0%) are dominant at the phylum level. At the class level, the relative abundance of bacteria on the leaves was ranked as follows: Gammaproteobacteria (34.4%), Alphaproteobacteria (26.6%), and Actinobacteria (23.2%) (see Figure 2 for a graphical representation). These findings highlight significant variations in the composition of bacteria among different tree species. Additionally, at the phylum level, the relative abundance of Proteobacteria was highest on the leaves of *F. mandshurica* (80.6%) and lowest on *T. amurensis* (46.2%). The relative abundance of Actinobacteria was highest on *T. amurensis* (40.9%) and lowest on *F. mandshurica* (14.2%). Moreover, the relative abundance of Firmicutes was highest on *A. mono* (4.2%) and lowest on *F. mandshurica* (0.1%).

### 2.2. Differences in Phyllosphere Bacterial Community among Tree Species

**Figure 3:**
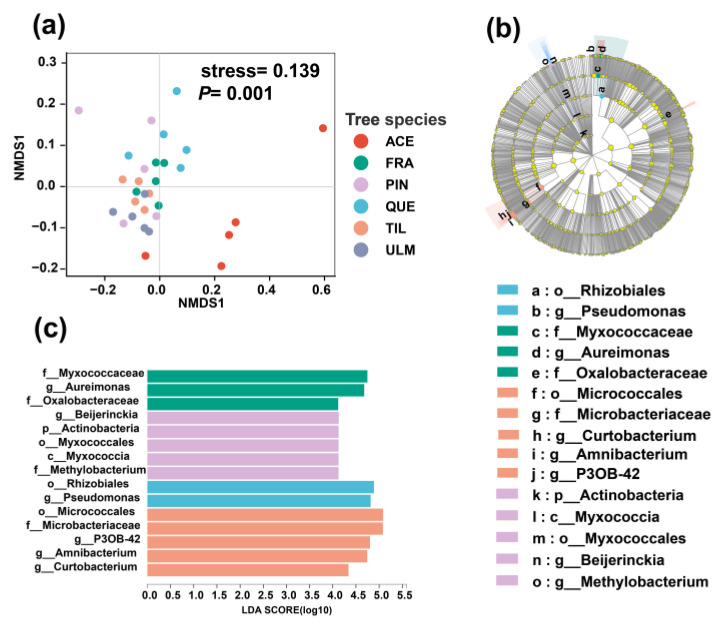
NMDS visualization diagram (**a**); cladogram of taxa which were differentially expressed based on Linear discriminant analysis Effect Size (LEfSe) (**b**); and Linear discriminant analysis (LDA) scores indicating the effect size of each differentially expressed taxon (**c**) of phyllosphere bacteria community structure of dominant tree species in a temperate mixed forest of Changbai Mountain. The stress value measures the goodness of fit of the NMDS solution to the original data. A lower stress value (closer to 0) indicates a better fit of the NMDS solution to the data, suggesting that the reduced-dimensional representation preserves the underlying structure of the original data. Abbreviations of tree species: ACE: *Acer mono*; FRA: *Fraxinus mandshurica*; PIN: *Pinus koraiensis*; QUE: *Quercus mongolica*; TIL: *Tilia amurensis*; ULM: *Ulmus japonica*.

ANOSIM analysis and NMDS showed that there are significant differences in microbial community composition among six tree species (Figure 3; Table 1). Based on the LEfSe analysis, the primary biomarkers for *F. mandshurica* were Myxococcaceae, *Aureimonas*, and Oxalobacteraceae. For *P. koraiensis*, the main biomarkers were Actinobacteria, Myxococcia, Myxococcales, *Beijerinckia*, and *Methylobacterium*. *Q. mongolica* had Rhizobiales and *Pseudomonas* as the primary biomarkers, while *T. amurensis* had Micrococcales, Microbacteriaceae, *Curtobacterium*, *Amnibacterium*, and P3OB-42 as the main biomarkers.

### 2.3. Correlations between Phyllosphere Bacterial Community and Leaf Functional Traits

Our study examined leaf functional traits across six plant species. Leaf area (LA), leaf thickness (SLA), leaf dry matter content (LDMC), leaf nitrogen content (LNC15), leaf carbon content (LCC), leaf calcium content (LCaC), leaf aluminum content (LAlC), leaf copper content (LCuC), leaf zinc content (LZnC), and leaf stomatal area (LSA) significant differences were observed among the plant species. Specifically, *Q. mongolica* exhibited the highest leaf area, measuring 65.368 cm^2^, while *P. koraiensis* displayed the lowest with a mere 0.730 cm^2^. *A. mono* had the highest carbon isotope composition, with a value of −27.789%., while *T. amurensis* had the lowest at −29.681%. *U. japonica* showed the highest leaf copper content at 5.111 g/kg, whereas *F. mandshurica* displayed the lowest content at 4.118 g/kg (Table 2).

**Figure 4:**
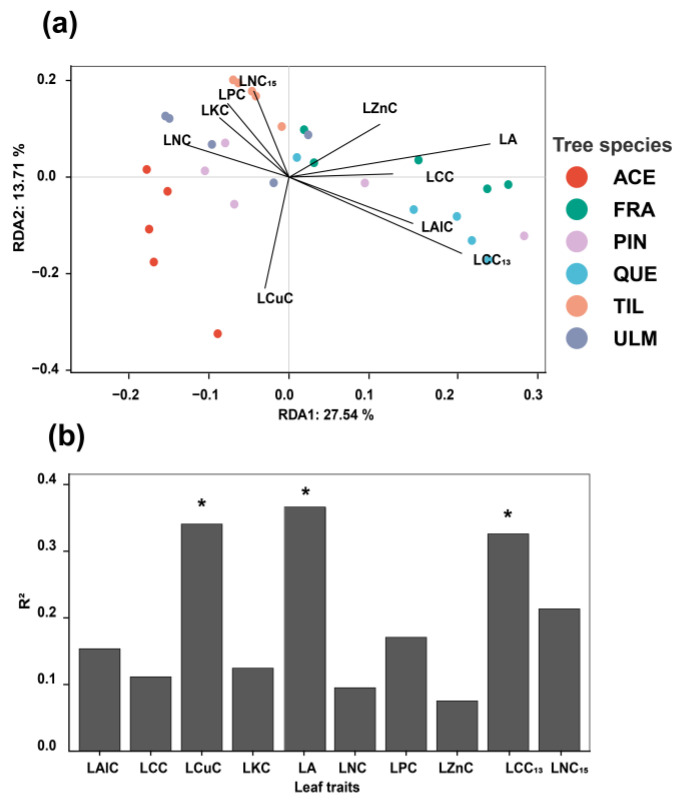
Redundancy analysis result (RDA) of phyllosphere bacterial community and leaf functional traits (**a**); and the explanatory power of each predictive factor (**b**). See Table 3 for the symbolic meanings of each factor in the figure; The asterisk (*) in the figure indicates that the factor has a significant influence on the microbial community (*p* < 0.05). Abbreviations of tree species: ACE: *Acer mono*; FRA: *Fraxinus mandshurica*; PIN: *Pinus koraiensis*; QUE: *Quercus mongolica*; TIL: *Tilia amurensis*; ULM: *Ulmus japonica*.

The redundancy and envfit analysis results showed that the functional traits relating to plant nutrient acquisition and resistance to diseases and pests (such as leaf area, isotope carbon content, and copper content) were the main factors influencing the community structure of phyllosphere bacteria (Figure 4).

### 2.4. Functional Genes of Phyllosphere Bacteria

**Figure 5:**
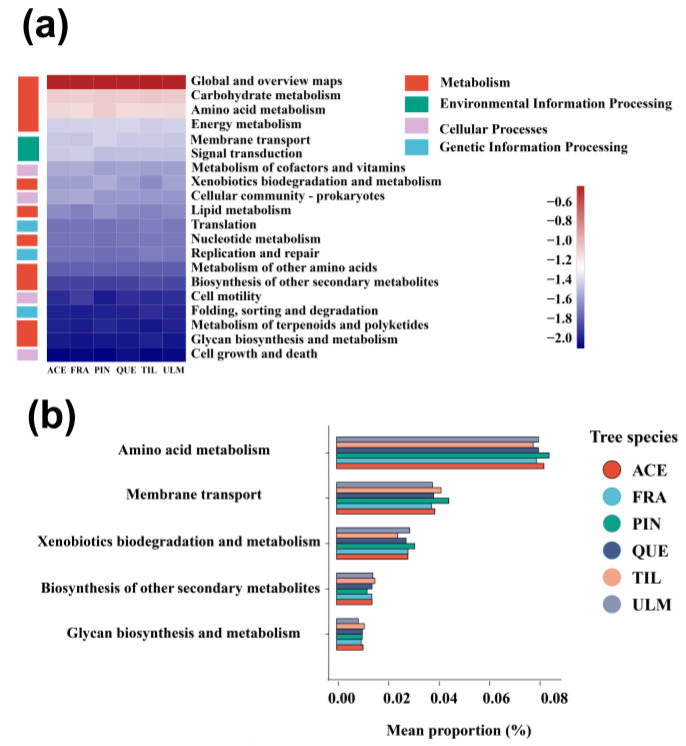
The function genes of phyllosphere bacteria of dominant tree species in a temperate mixed forest of Changbai Mountain. A heatmap illustrates the relative abundance of functions (**a**), and specifically in panel (**b**), only functions with statistical significance (*p* < 0.05) are presented in the comparison between tree species.Abbreviations of tree species: ACE: *Acer mono*; FRA: *Fraxinus mandshurica*; PIN: *Pinus koraiensis*; QUE: *Quercus mongolica*; TIL: *Tilia amurensis*; ULM: *Ulmus japonica*.

For the analysis of bacterial functions, the functions related to metabolism were the richest in our dataset, accounting for 76% of all functional annotation sequences (Figure 4a). The main metabolic processes were amino acid metabolism (9.1%), carbohydrate metabolism (8.4%), and energy metabolism (4.2%). Environmental and genetic information processing also had a high relative abundance, mainly membrane transport (3.9%), signal transduction (3.1%), and translation (2.1%) (Figure 5a). The variance test among tree species with secondary functions showed that there were significant differences in relative abundance among tree species with six metabolic processes including amino acid metabolism, membrane transport, xenobiotics biodegradation and metabolism, biosynthesis of other secondary metabolites, and glycan biosynthesis and metabolism. *P. koraiensis*’s amino acid metabolism and membrane transport were significantly higher than other tree species (Figure 5b).

## 3. Discussion

We found that tree species with closer evolutionary distances had more similar phyllosphere microbial diversity, which could be attributed to the assembly of host-associated microbiomes in long-term evolutionary processes [30,31]. Compared to tree species with smaller evolutionary distances, tree species with a long evolutionary distance may have generated greater selection pressure on the species pool of phyllosphere microbes, resulting in more significant differences in phyllosphere microbial diversity [32]. This finding is in line with the strong plant–bacterial interactions on leaves found in a neotropical forest in Panama [13], where most of the dominant bacterial taxa in the phyllosphere have significant evolutionary association with host tree species. These evolutionary associations are likely to be related to phylogenetic variation in host traits, given the interaction between microbial community variance explained by host traits and taxonomy [12].

Our results show that Proteobacteria, Actinobacteria, and Firmicutes were the dominant phyla observed in our samples. The dominant position of Proteobacteria is consistent with many other studies on the bacterial community composition of plant leaves [32,33,34,35,36]. This observation is attributed to the relatively faster replication rate of Proteobacteria compared to other phyla. The ability of Proteobacteria to replicate more rapidly may result in their higher abundance and dominance within the leaf microbiome, as they can colonize and proliferate more efficiently in the leaf environment [37]. Further, Proteobacteria have diverse metabolisms and perform important plant functions, such as nitrogen fixation, nitrification, methylation, and oxygen-free photosynthesis [38,39,40,41]. Actinobacteria and Firmicutes are typically associated with arid environments [40,41,42], which may explain their adaptability to thrive on leaf surfaces, often exposed to dry air and UV radiation [43,44,45]. 

Additionally, we found significant biomarkers for all tree species except *U.japonica* and *A. mono*. It is possible that the composition of the microbial community reflects how the relative microbial abundance responds to changes in the environment, according to studies by Liu et al. [30] and Maestre et al. [31]. This means that differences in microhabitats on the surface of leaves could be a factor in the unique microbial flora of different tree species. However, because *U. japonica* and *Acero mono* are usually in the secondary forest layer [46], they may be less affected by external environmental changes and overlap with the canopy layer and the primary forest layer, so there is no unique microbial flora [47]. Specifically, LEfse analysis shows that the *P. koraiensis* biomarker is Actinobacteria, primarily saprophytic bacteria, which produces enzymes that can degrade cellulose. One of the biomarkers for *P. koraiensis* is *Beijerinckia*, a free-living, nitrogen-fixing aerobic microorganism known for its abundant nitrogenase enzymes, which facilitate efficient nitrogen reduction [48]. Additionally, *Methylobacterium*, another biomarker for Korean pine, is recognized for its utilization of C1 compounds released by plants [49]. This shows that *P. koraiensis* may be essential in regulating forest climate and carbon and nitrogen cycles. Under drought stress, *Pseudomonas*, a biomarker of *Q. mongolica*, can produce extracellular polysaccharides to protect bacteria from water threats. Prior research has shown that the inoculation of *Pseudomonas* sp. into host plants can lead to a significant increase in the levels of proline, amino acids, and soluble sugars, ultimately bolstering the plants’ drought resistance [50]. *Q. mongolica*, a tree species renowned for its drought resistance in the Changbai Mountain area [51], may also derive potential benefits from the presence of Pseudomonas. The advantages that the presence of drought-resistant bacteria can bring to *Q. mongolica* include enhancing its ability to withstand water stress, facilitating the accumulation of osmoprotectants, and promoting overall resilience in challenging arid conditions [52]. Thus, the interaction with *Pseudomonas* could further contribute to the drought resistance observed in *Q. mongolica*. The natural products found in this family are expected to become an important source of drugs in the future [53]. This kind of bacteria is the biomarker of *U. pumila*, so the application value of *U. pumila* leaves should be further explored.

The redundancy analysis confirmed that certain traits are linked explicitly to phyllosphere microbial community composition. The carbon isotope content, leaf area, and copper in the leaves of plants have significant effects on the bacterial community in the canopy. Previous studies have suggested that leaf environmental conditions such as element concentration, resource availability, and defensive compounds can act as ecological filters and affect the microbial community composition on leaves [54]. Specifically, leaf area and leaf carbon isotope content mainly represent the resource acquisition ability of plants. Therefore, our findings show that the characteristics of the phyllosphere microbial composition of tree species are closely related to their nutrient acquisition ability [55]. In addition, leaf copper content is also essential to the composition of the phyllosphere bacterial community because they can also be used as a defensive compound for plants [40,56].

The abundance of functional genes affects the transformation of ecological processes by affecting microbial processes. The results of functional prediction support the critical role of carbohydrate and amino acid metabolism in the phyllosphere bacteria [57,58,59]. For example, an experimental study on the colonization of phyllosphere bacteria [58] showed that carbohydrate metabolism is produced during plant photosynthesis and is then consumed by other organisms, aiding the process of regulating the metabolic formation, decomposition, and mutual transformation of microbes [60]. Amino acid metabolism can help bacteria absorb amino acids, which is beneficial to the survival and reproduction of phyllosphere bacteria [61,62]. Membrane transporters are also reported as an important part of the functional library of epiphytic microbes, which can maximize the ability to monopolize other restricted resources [63]. The richness of signal transduction pathways involves rapid sensing and response to environmental changes, which will eventually be consistent with the high variability of humidity, light, and temperature conditions in the microbial habitat [17]. The variance analysis showed that amino acid metabolism and membrane transport of *P. koraiensis* was significantly higher than those of other tree species, which may imply that the phyllosphere bacteria of conifer species, *P. koraiensis,* had developed a stronger ability to absorb nutrients and transport energy due to their lower leaf area (i.e., insufficient nutrient acquisition), compared with broadleaf species. Generally speaking, phyllosphere bacteria in the broad-leaved Korean pine mixed forest can absorb more carbohydrates and amino acids from the leaves through these functional genes, which improves the diversity of phyllosphere bacteria. These potential biological functions lay the foundation for the interaction between plants and microbes. Phyllophere microbes may participate in many life processes of plants through these potential functions and help plants grow in nonideal conditions.

## 4. Materials and Methods

### 4.1. Study Site

The experimental plot is located in the broad-leaved Korean pine mixed forest (42°21′01″ N, 128°42′51″ E) of the National Nature Reserve in Changbai Mountain in northeastern China. The elevation ranges between 830 and 850 m within our study area. The study site has a typical temperate continental mountain climate, with an average annual temperature of 3.6 °C and average annual precipitation of 700 mm [64,65]. The zonal soil in this area is mountainous dark brown forest soil. The broad-leaved Korean pine mixed forest is rich in species composition and has a clear vertical structure. The dominant tree species include *Pinus koraiensis*, *Tilia amurensis*, *Quercus mongolica*, *Acer mono*, *Fraxinus mandshurica*, and *Ulmus japonica* [66]. 

### 4.2. Leaf Sampling

In August 2020, 6 dominant tree species (*P. koraiensis*, *T. amurensis*, *Q. mongolica*, *A. mono*, *F. mandshuric*, *U. japonica*) in broad-leaved Korean pine mixed forest were sampled by selecting 5 individual trees per species, with a total of 30 trees (i.e., 30 samples). The interval between each tree was greater than 20 m. We use averruncator to cut leaves from all four directions of the target tree species. To avoid the influence of leaf age and disease, we selected 20 healthy and undamaged leaves at the top of each branch, mixed them, and stored them at 4 °C until they could be returned to the laboratory to be kept at −80 °C. 

### 4.3. Measurements and Calculations of Leaf Functional Traits

Fourteen common leaf morphological and chemical traits related to plant life history and nutrient and water use efficiency were measured based on 5 to 10 leaves [67]. Leaf area (LA) was calculated using a portable scanner (Canon LiDE 110, Tokyo, Japan) and Image Pro Plus 6.0 software (Media Cybernetics, Silver Spring, MD, USA). Leaf dry matter content (LDMC) was determined after drying in a constant mass oven for 48 h at 65 °C. Specific leaf area (SLA) was calculated as the ratio of fresh leaf area to leaf dry matter content. The dried leaf samples were then ground to a fine powder using a ball mill (RETSCH, GmbH, Haan, Germany). Leaf carbon (LCC) and nitrogen (LNC) contents were measured using an elemental analyzer (Vario EL III, Elementar, Hanau, Germany). Leaf carbon isotope content (LCC13) and nitrogen isotope content (LNC15) were analyzed using a stable isotope analyzer (CMCRDS system, Picarro, CA, USA). Other chemical elements, including phosphorus (LPC), potassium (LKC), aluminum (LAlC), copper (LCuC), calcium (LCaC), and zinc (LZnC), were measured using an ICP Optima 8000 (Perkin-Elmer, Waltham, MA, USA). The size of the leaf stomatal area was measured using a nail polish blotting method [68]. The main functions of each of these traits are listed in Table 3.

**Table 3:** Plant traits and their corresponding functions used in this study.

Abbreviations	Functional Traits	Functions
LA	Leaf Area	Resource allocation capacity [69]
SLA	Specific Leaf Area	Resource allocation capacity [69]
LDMC	Leaf Dry Matter Content	Resource allocation capacity [70]
LCC_13_	Leaf Stable Carbon 13 Content	Water utilization efficiency [70]
LNC_15_	Leaf Stable Nitrogen 15 Content	Resource utilization efficiency [71]
LCC	Leaf Carbon Content	Plant photosynthesis [72]
LNC	Leaf Nitrogen Content	Plant photosynthesis [72]
LPC	Leaf Phosphorus Content	Plant photosynthesis [72]
LKC	Leaf Potassium Content	Plant photosynthesis [72]
LCaC	Leaf Calcium Content	Plant metabolism [73]
LAlC	Leaf Aluminum Content	Plant metabolism [73]
LCuC	Leaf Copper Content	Plant metabolism; resistance to diseases, pests [74]
LZnC	Leaf Zinc Content	Plant metabolism; resistance to diseases, pests [74]
LSA	Leaf Stomatal Area	Transpiration, photosynthesis [75]

### 4.4. Leaf DNA Extraction and Sequencing

For the extraction of phyllosphere bacteria, we ground 5–10 fresh leaves, weighed 5 g of them into sterile tubes, and added 10 mL of 0.1 M potassium phosphate buffer (pH = 8.0) to each gram of sample [12,14]. The samples underwent ultrasonic cleaning twice for 1 min and centrifuged for 10 s. The potassium phosphate buffers from the two washes were combined and filtered through a 0.2 μm sterile filter (Supor EAV, Pall Corporation, Ann Arbor, MI, USA). The filters were chopped, and DNA was extracted using the FastDNA^®^ SPIN Kit (Qbiogene, Irvine, CA, USA). DNA extracts were checked on a 1% agarose gel, and DNA concentration and purity were determined using a NanoDrop-2000 (Thermo Scientific, Wilmington, NC, USA). Sequencing was performed by Shanghai Meiji Biomedical Technology Co., Ltd. on the Illumina NovaSeq PE250 platform. The amplification primers were 799F (AACMGGATTAGATACCCKG) and 1193R (ACGTCATCCCCACCTTCC). Raw reads were saved to the NCBI database (SUB9956652).

### 4.5. Data Analyses

Raw gene sequencing reads were filtered using QIIME (version 1.7). Low-quality sequences (length < 200 bp, ambiguous bases > 0, average base quality score < 25) were removed. Samples were differentiated based on barcodes and primers, and sequence orientation was adjusted. UPARSE version 7.1 [76] was used to cluster sequences into operational taxonomic units (OTUs) with 97% similarity and to identify and delete chimeric sequences. The classification of the representative sequences of each OTU was species annotated by the RDP classifier [77] according to the Silva v138 bacterial database alignment. The alignment threshold was set to 80%, and 656,940 high-quality sequences were obtained. To avoid the influence of sequencing depth on subsequent analysis, all samples were diluted to the same sequencing depth (21,898 sequences per sample), resulting in 5231 OTUs.

We used the R package V.PhyloMaker [78] to construct a phylogenetic tree for six trees species. Based on the results of OTU cluster analysis, α-diversity (i.e., species richness) was calculated. ANOVA (Analysis of Variance) was used to explore whether α-diversity and leaf functional traits was significantly different among tree species. The Bray–Curtis matrix between samples was calculated at the OTU level and visualized by NMDS (Non-Metric Multidimensional Scaling) to depict differences in the bacterial community in a two-dimensional space. Significance was evaluated by ANOSIM (Analysis of Similarities) [79]. LEfSe analysis (Linear discriminant analysis Effect Size) was used to identify biomarkers causing differences in microbial community structure between groups using a Linear discriminant analysis (LDA) score threshold of 4.0 and an alpha level of 0.05 at the genus level and above on the Hutlab Galaxy website application (http://huttenhowe.sph.harvard.edu/galaxy/ 10 June 2023) [80]. The study utilized RDA (Redundancy Analysis) to investigate the relationship between the bacterial community in the canopy phyllosphere and leaf functional traits. The analysis also utilized the ‘envfit’ method to determine the explanatory power of each factor [77]. Finally, the functional annotation of PICRUSt2 predictions was obtained based on the Kyoto Encyclopedia of Genes and Genomes (KEGG) database [81]. Except for LEfSe analysis, other statistical analyses were conducted using R software (version 4.3.2). 

## 5. Conclusions

Our findings suggest that tree species with a close phylogenetic relationship tend to exhibit similar alpha phyllosphere bacterial diversity. Additionally, we observed multiple biomarkers for all tree species except *Ulmus japonica* and *Quercus mongolica*. The biomarkers of *Pinus koraiensis* may suggest that it is essential for forest climate as well as carbon and nitrogen cycles, and the biomarkers of *Quercus mongolica* may contribute to the drought resistance of the host. Our study also shows that the leaf traits of host tree species, such as leaf area, leaf carbon isotope content, and leaf copper and zinc contents could regulate the composition of the phyllosphere bacterial community. The results of functional prediction show that the main functional genes are carbohydrate metabolism and membrane transport, which can improve resource utilization capacity. The abundance of these two functions of *Pinus koraiensis* is higher than that of other tree species, which may be an evolutionary strategy of microbes in *Pinus koraiensis* under the condition of low leaf area. The results provide basic data for revealing the canopy biodiversity composition, structure, and driving factors of temperate forests and provide a useful reference for the study of plant-microbes interaction under global changes.

## Figures and Tables

**Table 1:** Analysis results of bacteria ANOSIM in leaves of dominant species of a temperate mixed forest in Changbai Mountain.

Comparison among Tree Species	R	*p*
Global	0.300	<0.001
ULM vs. ACE	0.216	0.025
ULM vs. TIL	0.228	0.023
ULM vs. PIN	0.504	0.010
ULM vs. FRA	0.636	0.008
ULM vs. QUE	0.796	0.008
ACE vs. TIL	0.260	0.016
ACE vs. PIN	0.480	0.020
ACE vs. FRA	0.356	0.016
ACE vs. QUE	0.312	0.025
TIL vs. PIN	0.524	0.009
TIL vs. FRA	0.792	0.011
TIL vs. QUE	0.696	0.010
PIN vs. FRA	0.540	0.011
PIN vs. QUE	0.488	0.007
FRA vs. QUE	0.660	0.011

**Table 2:** Leaf functional traits of six tree species ANOSIM in leaves of dominant species of the temperate mixed forest in Changbai Mountain. Asterisks indicate significant differences between tree species (* = *p*  <  0.05, ** = *p*  <  0.01, *** = *p* <  0.001). Abbreviations of tree species: ACE: *Acer mono*; FRA: *Fraxinus mandshurica*; PIN: *Pinus koraiensis*; QUE: *Quercus mongolica*; TIL: *Tilia amurensis*; ULM: *Ulmus japonica*.

	ACE (Mean ± SE)	FRA (Mean ± SE)	PIN (Mean ± SE)	QUE (Mean ± SE)	TIL (Mean ± SE)	ULM (Mean ± SE)
LA (cm^2^) ***	24.52 ± 1.69	36.08 ± 2.45	0.73 ± 0.035	65.36 ± 10.88	38.26 ± 3.86	17.02 ± 0.53
SLA (cm^2^/g) **	190.05 ± 30.52	260.03 ± 36.44	84.84 ± 11.57	310.555 ± 14.59	315.12 ± 20.76	76.56 ± 12.01
LDMC (g/g) **	0.092 ± 0.014	0.15 ± 0.04	0.02 ± 0.00	0.30 ± 0.04	0.17 ± 0.02	0.10 ± 0.01
LCC_13_ (d13C/12C) **	−30.58 ± 0.30	−27.78 ± 0.53	−30.51 ± 0.478	−29.47 ± 0.24	−29.68 ± 0.52	−29.32 ± 0.30
LNC_15_ (%) *	−2.39 ± 0.77	0.18 ± 0.67	0.48 ± 0.40	−0.20 ± 0.19	1.01 ± 0.49	−1.55 ± 0.44
LCC (%)	41.73 ± 0.09	42.34 ± 0.38	46.53 ± 0.70	43.04 ± 0.15	43.87 ± 0.38	39.30 ± 0.28
LNC (%)	1.96 ± 0.08	1.90 ± 0.26	1.604 ± 0.12	2.32 ± 0.08	2.09 ± 0.16	1.92 ± 0.11
LPC (g/kg)	1.87 ± 0.15	1.94 ± 0.49	2.10 ± 0.10	2.68 ± 0.26	3.03 ± 0.30	2.11 ± 0.11
LKC (g/kg)	0.07 ± 0.01	0.08 ± 0.00	0.04 ± 0.01	0.01 ± 0.00	0.08 ± 0.01	0.07 ± 0.01
LCaC (g/100 g) **	0.24 ± 0.01	0.25 ± 0.03	0.07 ± 0.02	0.139 ± 0.01	0.17 ± 0.02	0.30 ± 0.02
LAlC (g/kg) ***	0.63 ± 0.07	0.46 ± 0.07	1.63 ± 0.31	0.65 ± 0.14	0.45 ± 0.08	0.45 ± 0.06
LCuC (g/kg) **	4.90 ± 0.62	4.12 ± 0.52	4.77 ± 0.14	4.90 ± 0.62	3.44 ± 0.158	5.11 ± 0.50
LZnC (mg/kg) ***	63.99 ± 7.39	25.56 ± 1.15	64.49 ± 5.77	41.42 ± 8.66	30.34 ± 9.19	33.21 ± 5.33
LSA (10^−9^ m^2^) ***	7.37 ± 0.02	17.06 ± 0.05	12.96 ± 0.10	24.24 ± 0.07	7.90 ± 0.07	18.74 ± 0.09

## Data Availability

Microbe data are available from the NCBI Sequence Read ArchivAeArchive (SRA) database (Accession Number: PRJNA824213), other data that support the findings of this study are available from the corresponding author upon reasonable request.
